# A High-Throughput Cell-Based Screen Identified a 2-[(E)-2-Phenylvinyl]-8-Quinolinol Core Structure That Activates p53

**DOI:** 10.1371/journal.pone.0154125

**Published:** 2016-04-28

**Authors:** John Bechill, Rong Zhong, Chen Zhang, Elena Solomaha, Michael T. Spiotto

**Affiliations:** 1 Department of Radiation and Cellular Oncology, The University of Chicago, Chicago, Illinois, United States of America; 2 High-throughput Screening Facility, University of Illinois–Champaign Urbana, Champaign, Illinois, United States of America; 3 Biophysics Core Facility, The University of Chicago, Chicago, Illinois, United States of America; University of Michigan, UNITED STATES

## Abstract

p53 function is frequently inhibited in cancer either through mutations or by increased degradation via MDM2 and/or E6AP E3-ubiquitin ligases. Most agents that restore p53 expression act by binding MDM2 or E6AP to prevent p53 degradation. However, fewer compounds directly bind to and activate p53. Here, we identified compounds that shared a core structure that bound p53, caused nuclear localization of p53 and caused cell death. To identify these compounds, we developed a novel cell-based screen to redirect p53 degradation to the Skip-Cullin-F-box (SCF) ubiquitin ligase complex in cells expressing high levels of p53. In a multiplexed assay, we coupled p53 targeted degradation with Rb1 targeted degradation in order to identify compounds that prevented p53 degradation while not inhibiting degradation through the SCF complex or other proteolytic machinery. High-throughput screening identified several leads that shared a common 2-[(E)-2-phenylvinyl]-8-quinolinol core structure that stabilized p53. Surface plasmon resonance analysis indicated that these compounds bound p53 with a K_D_ of 200 ± 52 nM. Furthermore, these compounds increased p53 nuclear localization and transcription of the p53 target genes PUMA, BAX, p21 and FAS in cancer cells. Although p53-null cells had a 2.5±0.5-fold greater viability compared to p53 wild type cells after treatment with core compounds, loss of p53 did not completely rescue cell viability suggesting that compounds may target both p53-dependent and p53-independent pathways to inhibit cell proliferation. Thus, we present a novel, cell-based high-throughput screen to identify a 2-[(E)-2-phenylvinyl]-8-quinolinol core structure that bound to p53 and increased p53 activity in cancer cells. These compounds may serve as anti-neoplastic agents in part by targeting p53 as well as other potential pathways.

## Introduction

Many protein-protein interactions influence the oncogenic phenotype by regulating cell division, cell death and cell survival. In particular, oncogenes often alter p53 stability by directing p53 to ubiquitin conjugating protein complexes for proteasome-mediated degradation. For many cancers, amplification of the cellular E3 ubiquitin-protein ligase MDM2 (MDM2) leads to p53 ubiquitination and degradation [1]. In addition, viral oncoproteins such as the Human Papillomavirus (HPV) oncogene E6 directs p53 to the E6AP ubiquitin conjugating complex [2, 3]. To this end, many groups have focused anti-neoplastic approaches to target MDM2 or E6AP in order to restore p53 expression by inhibiting p53 degradation [4]. However, restoring p53 expression does not always equate to increased p53 activity. In chronic lymphocytic leukemia lines, tumor response to the MDM2 antagonist Nutlin depended on wild type p53 [5]. Therefore, cancer cells may acquire resistance to inhibitors of the p53-MDM2 interaction by defective p53 signaling, additional p53 mutations or compromised p53-dependent apoptosis [6].

Here, using a novel cell-based assay to screen for compounds that stabilize p53, we identified compounds that bound to and activated p53 as well as caused cell death. To develop this cell-based screen, we applied a targeted protein degradation strategy by targeting p53 to the Skip-Cullin-F-box (SCF) ubiquitin ligase complex. Our rationale for forcing p53 degradation through the SCF complex was to identify compounds that directly rescued p53 degradation rather than compounds that indirectly rescued p53 degradation by inhibiting MDM or E6AP pathways normally used to degrade p53. We controlled for compounds that did not target p53 by multiplexing this assay with a similar degradation assay for the retinoblastoma protein, Rb1, in order to avoid inhibitors of the SCF complex or other protein degradation pathways. Screening 158,000 compounds identified a class of compounds sharing a common 2-[(E)-2-phenylvinyl]-8-quinolinol core structure that rescued p53 degradation. These compounds bound p53 with a K_D_ of 200 ± 52 nM, activated the p53 pathway and caused cell death in multiple cancer cell lines.

## Material and Methods

### Cell culture, drugs and virus infection

HeLa and SiHa were obtained from the American Culture Collection and were a gift of Kenneth Alexander (The University of Chicago). Cervical cancer C33a cells and the breast cancer MCF7 cells were obtained from the American Culture Collection. Head and neck squamous cell cancer cell line SQ-20B were obtained from Dr. Ralph Weichselbaum [7]. HCT116 cells containing wild type p53 and HT116 cells containing a p53 null allele generated by Crispr targeted gene editing (GeneArt derived cell line) were obtained from LifeTechnologies. HeLa, SiHa and C33a cells were authenticated using IDEXX laboratories 9 loci STR testing. Cells were maintained in complete DMEM (cDMEM) with 10% fetal bovine serum plus L-glutamine and Penn/Strep at 37 degrees. The 158,000 compounds screened consisted of 8000 compounds from the NCI Open Plate Set and 150,000 compounds from Chembridge Microformat library. All studies were performed in accordance with the University of Chicago Institutional Biosafety Committee.

### Vector design

The following plasmids were obtained from addgene: GFP-p53 (Plasmid 12091, Tyler Jacks[8]) GFP-RB FL (Plasmid 16004, Olimpia Meucci[9]), pBeta-actin E6 E7 (Plasmid 13712, Karl Munger [10]), pDsRed-Max-N1 (plasmid 21718, Ben Glick[11]), MSCV-IRES-GFP (Plasmid 20672), MSCV IRES Luciferase (plasmid 18760, Scott Lowe), p4489 Flag-βTrCP (plasmid 10865, Peter Howley [12]). To generate the p53-Luc construct, the p53 gene was amplified from GFP-p53 using the p53 fwd primer and p53 rev primer (S1 Table) and was cloned into an EcoRI-XhoI digested fragment of MSCV-IRES-GFP to generate MSCV-p53-IRES-GFP. The Luc gene was amplified from the MSCV IRES Luciferase plasmid using the luc fwd primer and luc rev primer and the digested product cloned into the AgeI-XhoI sites of MSCV-p53-IRES-GFP to generate MSCV-p53Luc-IRES-GFP.

To generate the Rb-Ren construct, the truncated fragment, representing aa 379–927 containing the LXCXE domain that binds E7, was amplified from GFP-Rb using Rb fwd primer and Rb rev primer and the digested product cloned into an EcoRI-XhoI digested fragment of MSCV-IRES-GFP to generate MSCV-p60Rb-IRES-GFP. The Ren gene was amplified from pIS plasmid using the Ren fwd primer and the Ren rev primer and cloned into the AgeI -XhoI sites of MSCV-p60Rb-IRES-GFP to generate MSCV-p60Rb-Ren-IRES-GFP. The MSCV-p53Luc-IRES-GFP and MSCV-p60Rb-IRES-GFP were digested with EcoRI-NotI to liberate the p53-Luc-IRES-GFP and p60Rb-Ren-IRES-GFP and cloned into an EcoRI-NotI fragment of pacAd5-CMV-GFP to generate pacAd5-CMV- p53Luc-IRES-GFP and pacAd5-CMV- p60Rb-IRES-GFP.

The MSCV-IRES-dsRed containing a dsRed-Max gene replacing the GFP gene was digested with EcoRI-NotI to excise the IRES-dsRed and cloned into pacAd5-CMV-GFP to generate pacAd5-CMV-IRES-dsRed.To generate the βTrCP-E6, the N-terminus of βTrCP was amplified from the Flag-βTrCP plasmid using TRCP fwd primer and TRCP Rev and cloned the product into the EcoRI-BamHI site of pacAd5-CMV-IRES-dsRed to generate pacAd5-CMV-βTrCP -IRES-dsRed. The E6 fragment was amplified from pBeta-actin E6E7 using the E6 fwd primer and E6 rev primer and the product was cloned into BamHI-XhoI fragment of pacAd5-CMV-βTrCP -IRES-dsRed to generate pacAd5-CMV-βTrCP -E6-IRES-dsRed. To generate βTrCP -E7, the E7 fragment was amplified from pBeta-actin E6E7 using the E7 fwd primer and E7 rev primer and the product was cloned into BamHI-XhoI fragment of pacAd5-CMVb-TRCP-E6-IRES-dsRed to generate pacAd5-CMVb-TRCP-E7-IRES-dsRed.

### Adenoviral infection and high throughput screening

Adenoviruses were generated using the RAPAd CMV adenoviral expression system (Cellbiolabs, San Diego, CA). High-throughput screening was performed at the University of Illinois High-Throughput Screening Facility. For each group, 1x 10^7^ C33a cells were plated in 150 mm^2^ dishes in 20 ml cDMEM. For p53xTE6 group, 1 x 10^8^ GFU/mL of p53-Luc adenovirus and 1 x 10^9^ GFU/mL TE6 adenovirus were plated. For RbxTE7 group, 1 x 10^8^ GFU/mL of p60RbRen adenovirus and 1 x 10^9^ GFU/mL of βTrCP-E7 adenovirus were added. For control group, Ad-Red was substituted for TE6 or TE7 adenovirus in the p53 control and Rb control, respectively. 12-24h later, 10 μl cells were collected and plated in 384 well plates containing 10 μl DMEM with 20 μM compounds, making the final screening compound concentration of 10 μM, which is a concentration commonly used in cell-based high-throughput screens [13, 14]. Experimental wells were plated with 2000 p53-TE6 cells and 2000 Rb-TE7 cells per well. Control wells were plated with 2000 p53-Red cells and 2000 Rb-Red cells per well. After 8h, plates were stored at -20 C for 1–3 days and assayed using Dual Glo Luciferase Assay (Promega). p53-Luc counts were normalized based to the control Rb-Ren activity within individual wells. Positive hits were scored as ≥ 2-fold above vehicle treated p53-Luc wells.

### siRNA knockdown

C33a cells were transfected with 100 pmol siRNA for p53 (Dharmacon, L-003329-00-0005) or AllStar control (Qiagen) using lipofectamine2000 according to manufacturer’s instructions. The cells were incubated for 24 hrs and then split and incubated with 10 μM compounds. Cell viability was measured after 48 hours by Cell Titer Glo. Efficiency of knockdown was determined by quantitative RT-PCR using SYBR green.

### E6 degradation assay

For the *in vitro* assay, E6 was cloned into the pMAL vector in frame with the maltose binding protein. The E6 was expressed in bacteria and purified on amylose column. C33a cells were transfected with p53-Luc and incubated overnight. 50 ng recombinant E6 was incubated with 10 μM compound and C33a lysates for 6 hours at 30 degrees. We measured luciferase activity after 30 minutes. For the trypsin and chymotrypsin assay, C33a cells were incubated for 2 hours with the indicated compounds at 10 μM and assessed for chymotrypsin and trypsin activity using Proteasome-Glo assay (Promega). For the *in vivo* degradation assay, C33a cells expressing Ad-TE6 and Ad-p53 or expressing Ad-TE7 and Ad-Rb-Ren were treated for 8 hours with compounds. After incubation, cells were lysed and luciferase activity measured and p53 levels measured by Western blot.

### Cell viability and Annexin V/PI staining

For the viability assays, 20,000 C33a, HeLa, or siHA cells were plated in white 96 well flat bottom plates. The cells were treated with compounds at concentrations from 0.5 to 30 μM for 48 hrs. After incubation, Cell Titer Glo (Promega) was added to the cells and luminescence read in a Tecan II plate reader. For annexin V and PI staining, 500,000 C33a cells were treated with inhibitor for 24 hrs. The cells were stained with Annexin V-FITC and PI according to eBioscience manufacturer’s instructions and analyzed by flow cytometry.

### Western analysis and nuclear/cytoplasmic fractionation

For nuclear/cytoplasmic fractionation, 1 × 10^7^ C33a cells were treated with 30 μM compound for 6 hrs. The cells were washed with PBS and lysed in 1000 μL of cytoplasmic extraction buffer (10 mM KCl, 10 mM HEPES, pH 7.9, 0.1 mM EDTA, 1 mM dithiothreitol) and incubated on ice for 20 min. 50 μL of 10% NP-40 was added to each lysate, vortexed and then centrifuged at 12,000 × g for 10 min. The cytosolic fraction was collected and the pellet containing the nuclei suspended in 50 μL of nuclear extraction buffer (0.4 M NaCl, 20 mM HEPES, pH 7.9, 1 mM EDTA). The nuclear fraction was incubated for 30 min on ice. The samples were spun at 14,000 × g for 10 min and the supernatants were collected. To concentrate the cytoplasmic fraction, 500 μL methanol, and 200 μL chloroform were added and the samples were vortexed, centrifuged for 10 min at 14,000 × g, and the upper and lower layer were discarded. The solid interphase was collected and suspended in 50 μL of lysis buffer. Equal cell equivalents were loaded on a gel and electrophoresed. For whole cell lysates, C33a cells were lysed with a modified RIPA buffer and subjected to SDS-PAGE electrophoresis. The gels were transferred to nitrocellulose and blots probed with antibodies for p53 (Santa Cruz, antibody FL-303), lamin A/C, and calnexin (Santa Cruz Biotechnology).

### Immunofluorescence

C33a cells were plated into chamber slides and treated with 30 μM of compounds 3 or, 4 for 4, or 8 hours. As a control, 30 μM cisplatin was used. The cells were fixed with 2.5% formaldehyde for 30 minutes, followed by permeabilization with 0.01% Triton X100 for 30 minutes. Cells were blocked with 5% goat serum for 30 minutes and stained overnight with anti-p53 (Santa Cruz, antibody FL-303) or anti-phospho-H2A.X (cell signaling) in 1% FCS in PBS at 4°. The following day, the cells were washed with PBS and incubated for 45 minutes with anti-rabbit-FITC and DAPI in 1% FCS in PBS. The cells were washed and mounted with ProLong Gold.

### Molecular modeling

A list of compounds testing positive and negative in the structure activity relationship (SAR) were generated with a cLogP below 4.5. Drug Discovery Workbench was used to find the appropriate binding pockets and molecular docking on p53 (PDB: 1TUP) and E6 (PDB: 4GIZ). The compounds were individually molecular docked into the pockets allowing rotation of the flexible bonds for 1000 iterations. The binding scores and hydrogen bond scores of the different fits were then organized for each compound.

### Surface plasmon resonance

p53 or E6 (R&D systems) were suspended in 10 mM sodium acetate pH 5.0 and amine linked to a Biacore CM5 chip (GE systems). Free reactive amines were then blocked with ethanolamine. Compound 4 was diluted in HBS P buffer (GE systems) at concentrations 1–30 μM and run over the chip. The association time was monitored for 300s and disassociation time for 1000 sec. After each run, the chip was regenerated using 10 mM glycine pH 3.0. The sensograms were fit to a one-ligand model to determine the K_a_ and K_d_.

### Quantitative RT-PCR

RNA from C33a and HeLa was isolated by Trizol and reverse transcribed using the first strand synthesis kit from NEB. Primers for p53, GAPDH and PUMA were used for quantitative real-time PCR (qRT-PCR) [15]. Primer sequences include: PUMA (forward primer: CTCAACGCACAGTACGAG and reverse primer: GTCCCATGATGAGATTGTACAG), FAS (forward primer: TCTGCCATAAGCCCTGT and reverse primer: GTCTGTGTACTCCTTCCCT), p21 (forward primer: CCCTGGTTAGACCAAAGCCAT and reverse primer: GGCACGCCAAACAAATCTCC), and BAX (forward primer: TGGCAGCTGACATGTTTTCTGAC and reverse primer: TCACCCAACCACCCTGGTCTT). ΔΔCt values were determined for PUMA using GAPDH to normalize data. Bio-rad SYBR green was used for the amplification and detection of the PCR products on an iCycler (Bio-rad).

### Chromosomal Immunoprecipitation (ChIP)-qPCR

Cells treated with the indicated compounds for 8 hrs, harvested and ChIP was carried using a Chromatin Immunoprecipitation Assay Kit (EMD Millipore). p53-DNA complexes were immunoprecipitated using a mouse monoclonal DO-1 antibody (Santa Cruz) and incubated overnight at 4°C. qPCR amplifications was performed using the primer sequences Puma forward 5’- GCGAGACTGTGGCCTTGTGT, reverse 5’- CGTTCCAGGGTCCACAAAGT.

## Results

### A novel cell-based multiplexed assay for targeted degradation of p53 and Rb1

**Fig 1:**
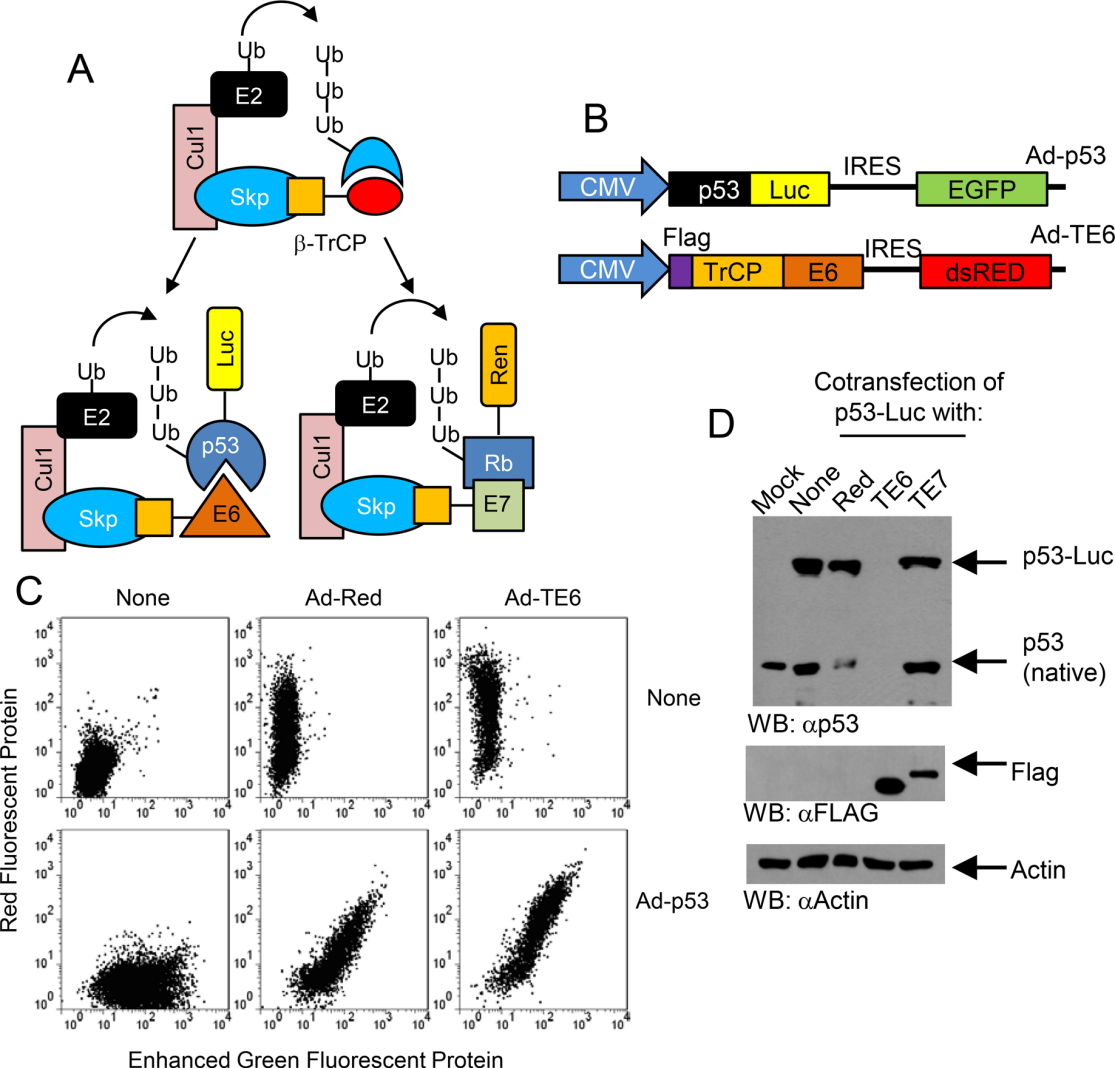
A multiplexed protein targeted degradation system to identify compounds that target p53. (A) Scheme for a protein targeted degradation strategy to monitor p53 degradation. The protein targeted degradation strategy was based on the Skp-Cullin-F-box (SCF) ubiquitin ligase complex which is an E3 ubiquitin ligase to which the adapter protein βTrCP binds with the F-box domain (orange square). βTrCP recruits target proteins to the SCF complex using its WD40 domain (red oval). When E6 (orange triangle) replaces the WD40 domain of βTrCP, the TE6 fusion protein recruits p53 for degradation. When E7 (green square) replaces the WD40 domain of βTRCP, the TE7 fusion protein recruits Rb for degradation. p53 and Rb which are fused to firefly (Luc) and renilla (Ren) luciferase reporters, respectively, to monitor protein levels. Inhibitors preventing the p53-E6 or Rb-E7 interaction would cause increased p53 or Rb fusion protein levels and their respective luciferase activities. (B) Scheme for adenoviral vectors Ad-p53 and Ad-TE6. Vectors contained an IRES-EGFP or IRES-dsRED as internal controls for p53 or TE6 expression, respectively. (C) TE6 did not affect EGFP expression that serves as an internal control for the p53-Luc reporter expression. C33a cells were infected with Ad-TE6, Ad-p53 or Ad-Red and analyzed for EGFP and RFP by flow cytometry. (D) TE6 expression caused loss of p53-Luc and endogenous p53 protein levels. C33a cells were infected with combinations of Ad-p53, Ad-TE6, Ad-Red and/or Ad-TE7. Whole cell lysates were assayed for p53 expression, TE6 expression using an anti-FLAG epitope or actin expression.

We developed a novel cell-based, multiplexed reporter system to screen for compounds that activate p53 using a previously described Skp-Cullin-F-box (SCF) complex targeted degradation approach [12, 16, 17]. The SCF complex is an E3 ubiquitin ligase that relies on βTrCP to target proteins for degradation. βTrCP consists of two separate domains, the F-Box domain that binds to the SCF complex and the WD40 domain that binds to its substrate (Fig 1A). Using this model, we targeted p53 to the SCF complex by fusing a flag tagged F-box domain of βTrCP to the E6 protein of the HPV-16 serotype to generate TE6. When TE6 binds to p53, it shuttles p53 to the SCF ubiquitin ligase complex resulting in p53 ubiquitination and proteasome degradation. To monitor p53 levels, we fused p53 to firefly luciferase creating the p53-Luc gene. For a specificity control, we fused E7 to βTrCP (TE7) and fused Rb to renilla luciferase (Rb-Ren).

We generated adenoviral vectors containing the TE6 cassette followed by an internal ribosomal entry site (IRES) driven dsRED fluorescent protein to monitor cells expressing TE6 (Fig 1B). As a control for p53 degradation, the p53-Luc gene followed an IRES-Enhanced Green Fluorescent Protein (EGFP) gene that served as an internal control for p53-Luc transcription in the presence of p53-Luc protein degradation. Fluorescence cytometry of cells expressing TE6 and p53-Luc demonstrated that the levels of dsRED fluorescence, a proxy of TE6 expression, were directly proportional to the levels of EGFP fluorescence, a proxy of p53-Luc expression. Therefore, higher levels of TE6 expression, which was designed to modulate the post-translational levels of p53-Luc, did not affect p53-Luc transcription or translation as measured by EGFP fluorescence (Fig 1C). Using Western analysis, we observed that p53 protein expression was decreased in cells infected with TE6 but not TE7 or dsRed indicating that TE6 caused the degradation of p53-Luc protein (Fig 1D). Thus, we have developed an assay to monitor the post-translational regulation of p53 by the viral oncoprotein E6.

### High-throughput screening for compounds that restored p53 reporter activity

**Fig 2:**
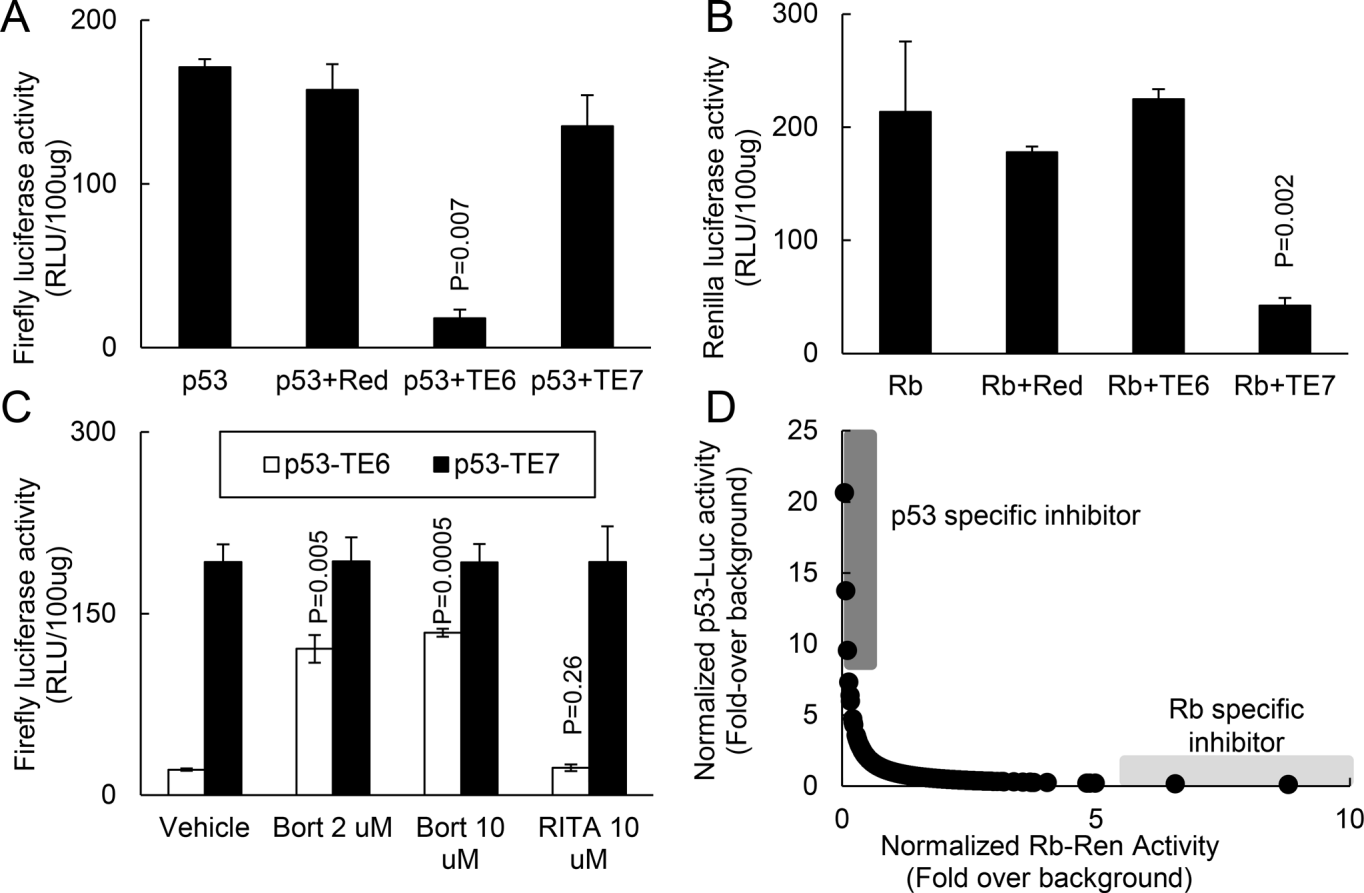
High-throughput screen for compounds that stabilized the p53 reporter assay. (A) TE6 abrogated p53-Luc luciferase activity. C33a cells were infected with Ad-p53-Luc alone or with Ad-Red, Ad-TE6 or Ad-TE7. (B) TE7 abrogated Rb-Ren luciferase activity. C33a cells were infected with Ad-Rb-Ren alone or with Ad-Red, Ad-TE6 or Ad-TE7. (C) TE6 promoted proteasome degradation of the p53-Luc reporter. C33a cells infected with Ad-p53-Luc and the cognate Ad-TE6 or non-cognate Ad-TE7 and treated with different concentrations of the proteasome inhibitor bortezomib or the p53 inhibitor RITA. (D) High throughput screening using the TE6-p53-Luc reporter detected compounds that restored p53 activity. A plot of 465 compounds selected from 158,000 compounds that induced p53-Luc or Rb-Ren activity at least 2-fold above background. p53-Luc activity was normalized to the corresponding Rb-Ren activity and vice versa to account for potential proteasome inhibitors or compounds with cytotoxic activity. The dark grey area indicates compounds that restored p53-Luc activity; the light grey area indicates compounds that restored Rb-Ren activity. Paired t-tests were used to determine significance.

To apply the p53 targeted degradation strategy to high-throughput screening, we developed a multiplexed assay to simultaneously monitor the stability of p53 and Rb. Analogous to Fig 1, we also generated adenoviral vectors to express TE7 and Rb-Ren fusion proteins (data not shown). For our cell based screen, we used C33a cells that expressed high levels of a p53 containing an Arg → Cys at amino acid 273. Since C33a cells expressed high levels of its endogenous p53^R273C^, C33a cells enabled the p53-Luc reporter to be expressed at high levels and not subjected to increased degradation by MDM2 or E6AP pathways. To confirm that TE6 degraded p53-Luc activity, we infected C33a cells with Ad-p53-Luc alone or with Ad-TE6 or the control vectors Ad-Red or Ad-TE7. p53-Luc luciferase activity was decreased only in cells also expressing TE6 but not in mock infected cells or in cells infected with the control vectors (Fig 2A). By contrast, cells expressing Rb-Ren had decreased luciferase levels only in cells expressing Ad-TE7 but not in control cells (Fig 2B). To determine if TE6 modulated p53-Luc activity via proteasome degradation, we treated C33a cells expressing p53-Luc and TE6 or TE7 with the proteasome inhibitor bortezomib. Bortezomib restored p53-Luc luciferase activity in cells expressing TE6 but not in control cells expressing TE7 (Fig 2C). Since bortezomib only partly restored p53-Luc activity in cells also expressing TE6, the inability to fully rescue p53-Luc degradation may have decreased the sensitivity of our assay. Furthermore, RITA, a putative inhibitor of the p53-MDM2 and p53-E6AP interactions [18, 19], did not restore p53-Luc activity suggesting that our protein targeted degradation strategy relied on TE6 targeting p53-Luc to the SCF complex. Therefore, we have developed a novel system that monitors p53 degradation and controls for non-specific mechanisms of p53-Luc stabilization. Furthermore, we have developed a multiplexed assay for high-throughput screening of p53 stabilization in order to avoid identifying inhibitors targeting SCF-degradation or other proteolytic pathways.

Using this reporter system, we screened 158,000 compounds from two different chemical libraries. In a single well, we plated C33a cells infected with Ad-TE6 and Ad-p53 (TE6-p53) with C33a cells infected with Ad-TE7 and Ad-Rb (TE7-Rb). We treated each well with individual compounds and subsequently assessed firefly and renilla luciferase activities. We identified 465 compounds that restored p53-Luc and/or Rb-Ren activity ≥ 2-fold above the untreated control. To avoid compounds that non-specifically blocked protein degradation and to account for any cytotoxic effects, we normalized p53-Luc activity to Rb-Ren activity and vice-versa (Fig 2D). Using these criteria, we identified 269 compounds that restored p53-Luc activity.

### A common 2-[(E)-2-phenylvinyl]-8-quinolinol core structure inhibited p53 degradation *in vitro*

**Fig 3:**
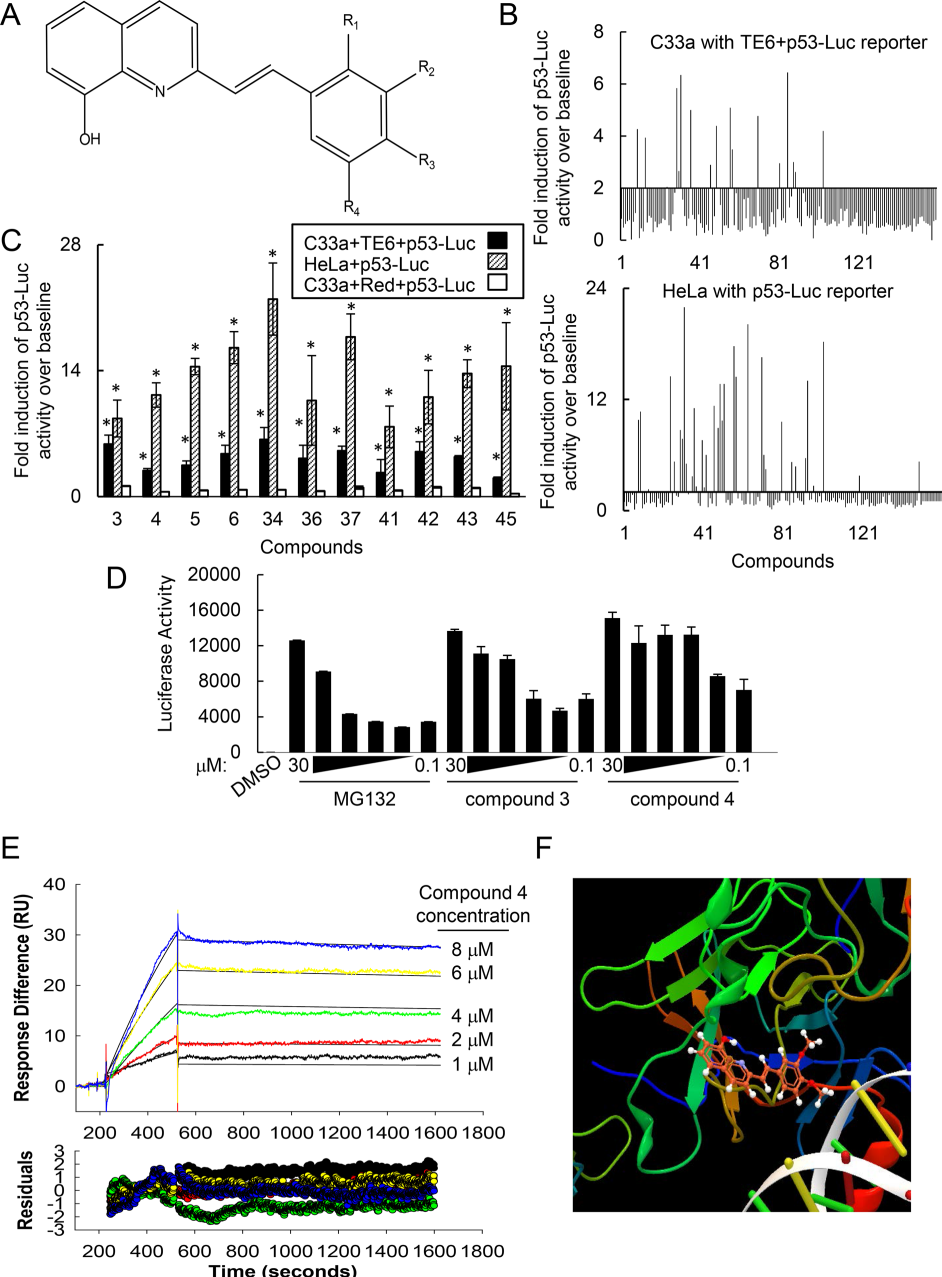
The common 2-[(E)-2-phenylvinyl]-8-quinolinol core structure bound p53 and disrupted p53 degradation. (A) Of the 269 potential compounds that increased p53-Luc activity by ≥2-fold above background, 6 structures shared common core 2-[(E)-2-phenylvinyl]-8-quinolinol structure. (B) Structure activity relationship studies identified additional compounds containing a core 2-[(E)-2-phenylvinyl]-8-quinolinol structure that disrupted p53 degradation. Upper panel: Activity of compounds in C33a cells expressing TE6 and p53-Luc. Lower panel: Activity of compounds in HeLa cells expressing p53-Luc. (C) 2-[(E)-2-phenylvinyl]-8-quinolinol compounds rescued p53 degradation. The top 11 compounds in (B) were assayed for restoration of p53-Luc activity in C33a cells expressing TE6, in HeLa cells or in C33a cells expressing dsRed. * indicates P < .001. (D) Compounds containing the core structure restored p53 activity *in vitro*. Recombinant E6 was incubated with the indicated compounds as well as with cell lysates containing p53-Luc protein. (E) Surface plasmon resonance demonstrated that compound 4 bound to p53 with a K_D_ of 200 + 52 nM. p53 recombinant proteins were amino terminally linked to a Biacore chip. Compound 4 was run at concentrations from 1–25 μM over the Biacore chip and association and disassociation times recorded. (F) Compound 4 docks with the DNA binding domain pocket of p53. Molecular modeling was performed with MolDock.

After examination of the structures, we identified 6 compounds containing a common 2-[(E)-2-phenylvinyl]-8-quinolinol core structure (Fig 3A; S2 Table). To confirm the activity of this core structure, we obtained 160 compounds that were two or three-dimensional analogues of the initial hits containing the common 2-[(E)-2-phenylvinyl]-8-quinolinol core structure. We tested these additional core structures on the restoration of luciferase activity in C33a cells expressing both TE6 and p53-Luc. 24 of 160 compounds induced p53-Luc activity ≥ 2-fold above background in C33a cells expressing both TE6 and p53-Luc (Fig 3B). Furthermore, in HeLa cells expressing an endogenous E6 that degraded p53-Luc, 36 of these 160 compounds increased p53-Luc activity at least 2-fold above background (Fig 3B). Therefore, these compounds prevented p53 degradation in cells endogenously expressing E6. S2 Table shows the top 24 compounds that were active in C33a and/or HeLa reporter assays. Fig 3C demonstrates the activity of the top 11 compounds in C33a cells expressing TE6 and p53-Luc, HeLa cells expressing p53-Luc or control C33a cells expressing Red and p53-Luc. Compounds that induced p53-Luc activity shared similar physical properties consistent with Lipinski’s rule of 5: cLogP values less than 5, molecular weight less than 500 and less than 5 H bond donors. Based on their ability to restore p53-Luc activity, we selected compound 3 and compound 4 to be used in further analyses (S2 Table).

To assess the impact of the common 2-[(E)-2-phenylvinyl]-8-quinolinol core structure on p53 degradation, we tested the ability of compounds 3 and 4 to restore p53-Luc activity using *in vitro* degradation assays. Core structure compounds 3 and 4 restored p53 levels *in vitro* to similar levels as the proteasome inhibitor MG132 (Fig 3D). Thus, we have identified compounds with a common 2-[(E)-2-phenylvinyl]-8-quinolinol core structure that inhibited p53 degradation.

### The 2-[(E)-2-phenylvinyl]-8-quinolinol core structure bound to p53

In order for p53 to be degraded in our reporter assay, these compounds likely bound to either p53, E6 or the adaptor protein E6AP. Consequently, we used Surface Plasmon Resonance (SPR) to assess binding of the 2-[(E)-2-phenylvinyl]-8-quinolinol core compounds between 1 to 25 μM concentrations to recombinant p53, E6 or E6AP. SPR analysis indicated that compound 4 bound to p53 with a K_D_ of 200 ± 52 nM (Fig 3E). By contrast, compound 4 bound to E6 with 16-fold lesser affinity (K_D_ 3.2 ± 1 μM) and did not have detectable affinity for E6AP (data not shown). To confirm that these compounds bound p53, we assessed the fit of the core structure compounds with the p53 core domain binding pocket adjacent to the DNA binding domain. Indeed, compound 4 fit into the binding pocket of p53 and all core compounds formed hydrogen bonds with the p53 core domain. (Fig 3F; S3 Table). This DNA binding domain is important for p53 function and is required for both E6 and MDM2-mediated degradation [20]. Thus, our data indicates these core compounds bound p53.

### Compounds activated p53 cell death pathways in cancer cell lines

**Fig 4:**
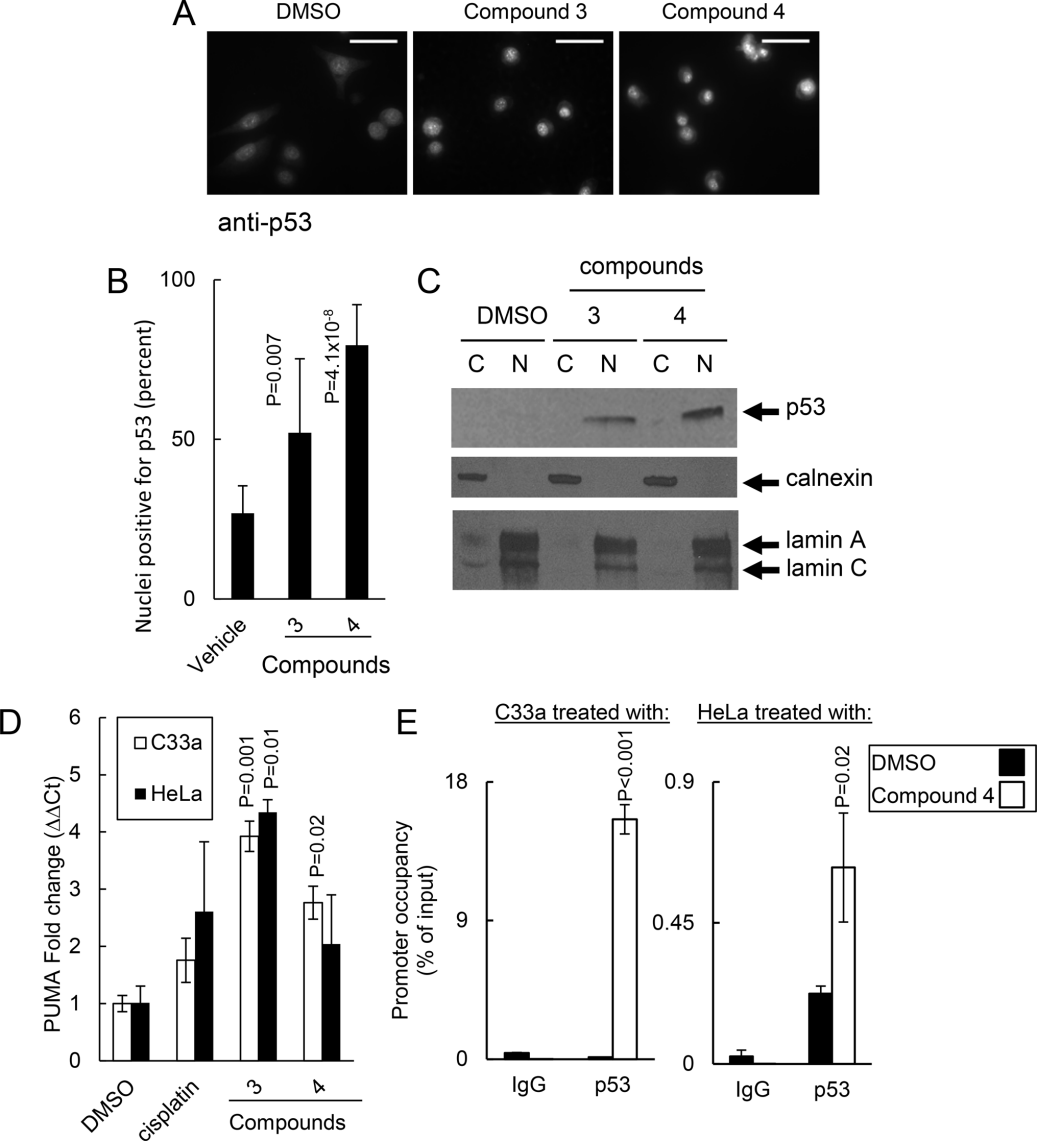
Compounds caused the p53 nuclear localization and target gene expression. (A) Compounds 3 or 4 induced p53 nuclear localization. C33a cells were treated with 30 μM compounds for 8 hrs and p53 localization was assessed by immunofluorescence. Representative images from 2 experiments taken at 40X magnification, scale bar = 20 μM. (B) Quantification of p53 nuclear accumulation in (A). (C) Compounds induced p53 protein accumulation in the nucleus. C33a cells were treated with indicated compounds and 6 hrs later fractionated by differential centrifugation. Cytosolic and nuclear fractions were assessed for p53 protein using Western analysis. Calnexin and Lamin A/C mark the cytosolic and nuclear fractions, respectively. (D) Core compounds induced transcription of the p53 target gene PUMA in cancer cells. C33a or HeLa cells were treated with the indicated compounds and, 8h later, PUMA expression as assessed by qRT-PCR using ΔΔCT for GAPDH normalization. (E, left panel) Compound 4 induced p53 binding to the PUMA promoter in C33a cells. (right panel) Compound 4 induced p53 binding to the PUMA promoter in HeLa cells. Results represent experiments performed in triplicate. Significance was assessed using the Student’s t-test.

Given that these core compounds bound p53 and inhibited p53 degradation, we assessed the extent to which compounds impacted p53 activation. We assessed p53 localization in C33a cells to confirm that these core compounds activated the p53 pathway. Upon activation, p53 translocates to the nucleus and binds to the promoters of p53 responsive genes. To determine if core compounds stimulated p53 nuclear localization, we treated C33a cells with 30 μM of compounds 3 and 4. After 8 hrs, we observed that p53 accumulated significantly more in the nucleus of cells treated with compounds 3 or 4 compared to vehicle alone (51.1±2.18% of cell nuclei for compound 3, P = .007; 79.5±12.7% of cell nuclei for compound 4, P = .009; 26.8±8.1% of cell nuclei for vehicle alone; Fig 4A and 4B). To confirm nuclear accumulation of p53, we treated C33a cells with compounds and fractionated the cytosolic and nuclear compartments. Cells treated with either compound 3 or 4 had higher levels of nuclear accumulation of p53 compared to vehicle-treated cells (Fig 4C). Since compounds induced p53 nuclear accumulation, we assessed the impact of compounds on transcription of the p53 target gene PUMA. After 8h of treatment with 30 μM, compounds 3 and 4 upregulated PUMA transcription 3.92±0.27-fold and 2.76±0.29-fold, respectively, in C33a cells and upregulated PUMA transcription 4.35±0.22-fold and 2.04±0.85-fold, respectively in HeLa cells (Fig 4D). To confirm that core compounds induced transcription of other p53 target genes, we treated HeLa cells with compound 3, 4, 37 or 38 and assessed the changes in expression in the p53 target genes p21, BAX and FAS (S1 Fig). Cells treated with these core compounds induced expression of p21, BAX and FAS consistent with the model that these compounds activated p53. To confirm that PUMA transcriptional activation was p53-dependent, we performed ChIP-qPCR on C33a cells and HeLa cells treated with compound 4. Treatment of cells with compound 4 induced p53 binding to the PUMA promoter by 38.9±2.4-fold (P < .001) and 31.3±8.7-fold (P = .02) for C33a and HeLa cells, respectively, compared to controls (Fig 4E). Thus, these core compounds activated the p53 pathway as evidenced by inducing nuclear accumulation of p53, binding of p53 to a target gene promoter and inducing transcription of p53 target genes.

### The 2-[(E)-2-phenylvinyl]-8-quinolinol core structure was cytotoxic to cancer cells

**Fig 5:**
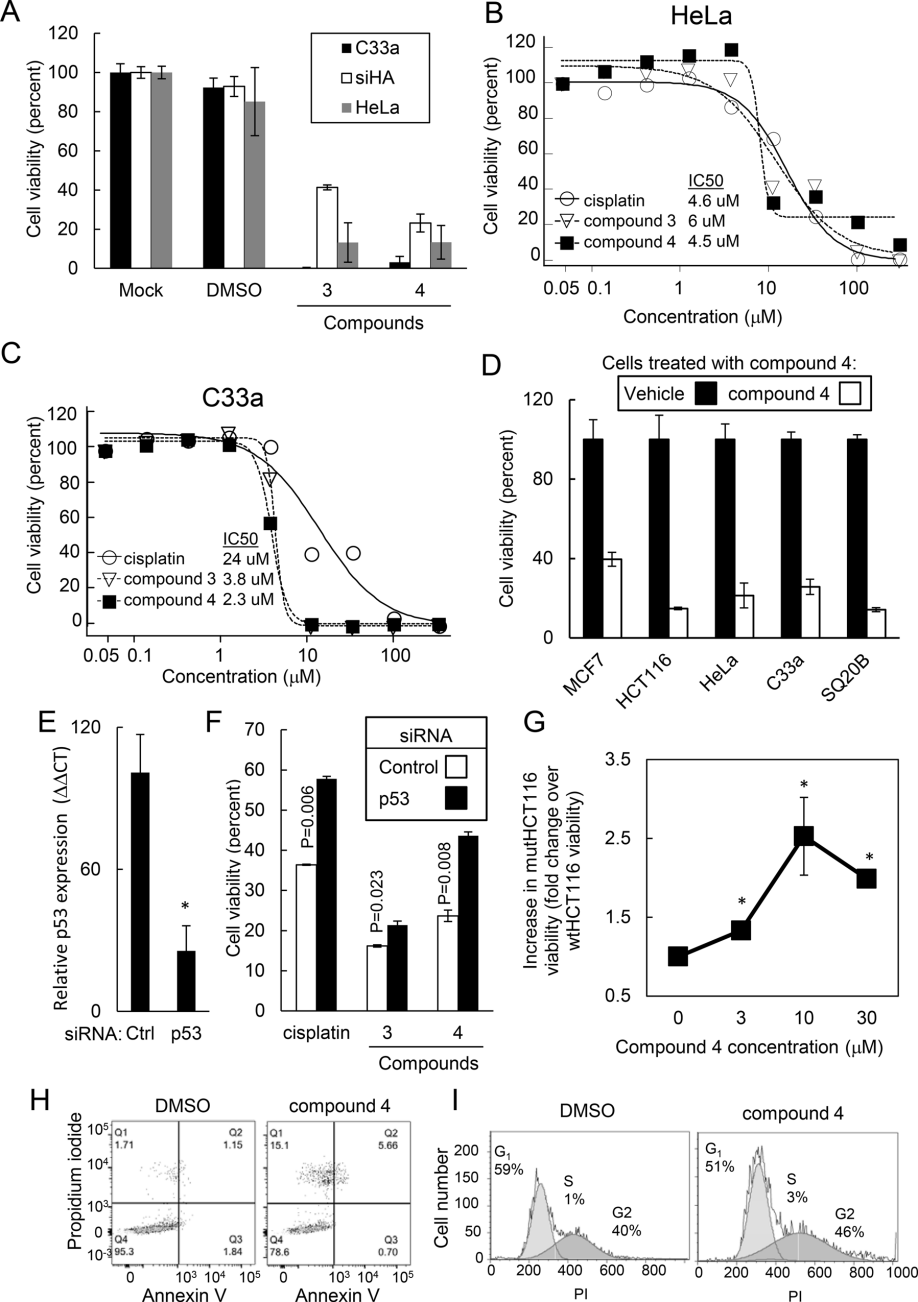
Common core structures were cytotoxic to cancer cells in part through a p53-dependent mechanism. (A) Compounds sharing the common core structure caused decreased viability in the cancer cell lines HeLa, siHA, and C33a. Cells were treated for 48h with compound 3 and 4 at 30 μM concentrations and viability was assessed with Cell Titer-Glo. (B-C) Core structures are cytotoxic with IC_50_ less than 10 μM. HeLa (B) and C33a (C) cells were incubated with the indicated compounds or cisplatin for 48 hrs and viability measured by Cell Titer Glo. IC_50_ was determined. (D) Compound 4 caused decreased viability in MCF7, HCT116, HeLa, C33a and SQ20B cells. (E) siRNA inhibition of p53 expression in C33a cells. Cells were transfected with siRNA targeting p53 or a control scrambled sequence and, 48h later, total RNA was assessed for p53 expression. (F) p53 knockdown abrogated the cytotoxicity of compounds containing the common 2-[(E)-2-phenylvinyl]-8-quinolinol core structure. C33a cells were transfected with siRNAs targeting p53 or a controlled scrambled sequence and, 24h later, were treated with 10 μM of the indicated compounds for 48 hrs. Viability was assessed using Cell Titer Glo. (G) Compound 4 was more toxic to wtHCT116 cells possessing a wild type p53 compared to mutHCT116 possessing a null p53 allele. Data was represented as the percent cell viability of mutHCT116 cells divided by the percent cell viability of wtHCT116 cells. * indicates P value < .01 (H) Compound 4 caused cell death as measured by Annexin V and PI staining. (I) Compound 4 did not affect the cell cycle as assessed by PI staining.

Given that the 2-[(E)-2-phenylvinyl]-8-quinolinol core structure activated the p53 pathway, we assessed the impact of core compounds on the growth of various cancer cell lines *in vitro*. We used cancer cell lines with the following alterations in p53 activity: (1) HeLa and siHA cervical cancer cells that degraded wild type p53 by expression of the viral oncogene E6;(2) C33a cervical cancer cells that overexpressed a mutant p53^R273C^ protein; (3) MCF7 breast cancer cells possessing a wild type p53; (4) HCT116 (wtHCT116) colon cancer cells possessing a wild type p53; (5) HCT116 (muttHCT116) cells with a homozygous p53 deletion caused by Crispr gene editing and (6) SQ20B head and neck cancer cells expressing a mutant p53. After treating HeLa, siHA, and C33a cells with 30 μM of compounds for 48h, the core structures caused decreased cell viability (Fig 5A). To assess sensitivity of cancer cells to these compounds, we determined the IC_50_ for specific cell lines. Compounds 3, 4 and cisplatin had an IC_50_ of 6, 4.5 and 4.6 μM, respectively, for HeLa cells (Fig 5B). Compounds 3 and 4 and cisplatin had an IC_50_ of 3.8, 2.3 and 24 μM, respectively, for C33a cells (Fig 5C). In addition, we also assessed the cytotoxicity of compounds in other cell types possessing wild type or mutant p53. Compounds were similarly cytotoxic for cells with wild type p53 (MCF7, wtHCT116 and HeLa) and for cells with mutant p53 (C33a and SQ20B; Fig 5D). Since core compounds activated the p53 pathway, we assessed if the cytotoxicity of core compounds to C33a cells was mediated by the p53 pathway. We used siRNA knockdowns to inhibit p53 expression in C33a cells. C33a cells transfected with p53 siRNA expressed p53 mRNA at 4.7±2.2-fold lower levels compared to C33a cells transfected with the scrambled siRNA control as assessed by qRT-PCR (Fig 5E). When cells were treated with 10 μM of compound 3 and 4, p53 gene knockdowns significantly reversed C33a sensitivity to the core compounds (Fig 5F). To confirm that the cytotoxicity of compounds was in part mediated by p53, we treated mutHCT116 cells possessing p53-null alleles and wtHCT116 cells possessing wild type p53 alleles with compound 4 and assessed cell viability (Fig 5G and S2 Fig). At 10 μM concentration of compound 4, mutHCT116 cells were 2.5±0.5-fold more viable than wtHCT116 cells (P = 0.006). However, loss of p53 did not completely restore cell viability suggesting that these compounds targeted both p53-dependent and p53-independent pathways. We further assessed cell death using annexin V and propidium iodide (PI) staining on C33a cells treated with compound 4. Compound 4 increased both the number of apoptotic and necrotic cells (Fig 5H). By contrast, cells treated with compound 4 had a normal cell cycle distribution indicating that these compounds did not affect the cell cycle (Fig 5I). Therefore, core compounds were cytotoxic to cancer cells partly through a p53-dependent mechanism; however, loss of p53 did not completely rescue cells from the cytotoxic effects of core compounds suggesting that compounds may target both p53-dependent and p53-independent pathways.

Given that p53 loss only partially rescued the growth suppression of core compounds in mutHCT116 cells, we assessed if these compounds also activated p53 related family members p73 and/or p63. Western analysis detected p73 in the nuclear lysates of mutHCT116 cells (S3 Fig) but did not detect p63 expression (data not shown). Treatment of mutHCT116 cells with compound 4 did not increase p73 nuclear accumulation suggesting that the toxicity of core compounds was unlikely due to activation of p53-related family members p73 and/or p63.

### The 2-[(E)-2-phenylvinyl]-8-quinolinol core structure did not cause genotoxicity or inhibit proteasome activity

**Fig 6:**
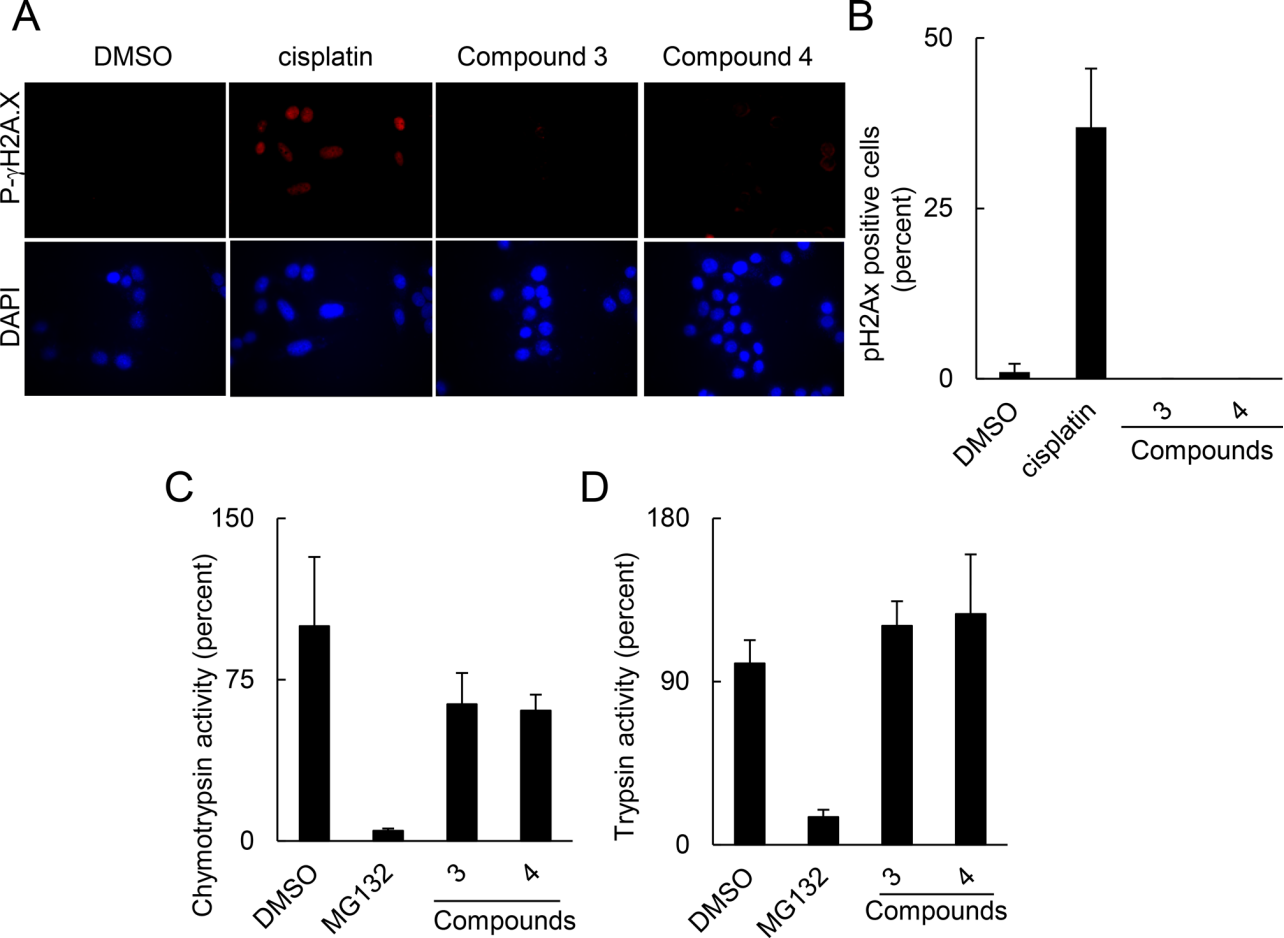
The common 2-[(E)-2-phenylvinyl]-8-quinolinol core structure did not cause DNA damage or inhibit proteasome activity. (A) Compounds did not cause double stranded DNA damage as measured by γH2AX phosphorylation. C33a cells were treated with 30 μM of compounds or cisplatin for 4 hrs, fixed and stained for phosphorylated γH2AX. (B) Quantitation of phosphorylated γH2AX positive nuclei. Results are from 10 fields per 2 replicates of the indicated condition. Impact of compounds on proteasome activity as measured by chymotrypsin-like activity (C) or trypsin-like activity (D). For (C) and (D), C33a cells were incubated for 2 hrs with the indicated compounds at 10 μM or 30 μM and assessed for chymotrypsin and trypsin activity using Proteasome-Glo assay. Paired T test were used to determine significance.

p53 expression may also be increased by causing genotoxicity that induces p53 transcription or by inhibiting proteasome degradation. To determine if these compounds damaged DNA, we treated cells with cisplatin, compounds 3 or 4 and assessed phosphorylated γH2AX that serves as a marker for DNA damage. Compounds 3 and 4 resulted in 0.49±0.585 and 0.44±0.89% of cells with phosphorylated γH2AX that was not significantly different from vehicle treated cells (1.01±1.24% of cells with phosphorylated γH2AX; Fig 6A and 6B). By contrast, cisplatin significantly increased γH2AX phosphorylation (36.92±8.64% of cells with phosphorylated γH2AX; P = 8.5 x 10^−5^). In addition, the proteasome possesses trypsin-like and chymotrypsin-like activities that mediate protein degradation. Using bioluminescent probes to interrogate trypsin-like and chymotrypsin-like activities, we assessed the impact of compounds 3 and 4 on proteasome degradation. Compounds 3 and 4 did not significantly reduce chymotrypsin-like activity (P = .09 for compound 3 and P = .13 for compound 4; Fig 6C) or trypsin-like activity (P = .33 for compound 3 and P = .12 for compound 4; Fig 6D). By contrast, MG132 significantly inhibited both chymotrypsin and trypsin activities. Thus, our data suggests that the core structure compounds did not restore p53 by non-specifically blocking proteasome degradation or by inducing DNA damage.

## Discussion

Here, we developed a novel, multiplexed protein targeted degradation assay to identify compounds that increased p53 stability. The screen allowed us to rapidly identify cell permeable compounds that increased p53 reporter levels in the presence of targeted degradation. The tandem Rb reporter allowed us to eliminate compounds which non-specifically increased E6-p53 reporter activity and to avoid compounds that would potentially target SCF- or proteasome-mediated degradation. In doing so, we identified compounds with a 2-[(E)-2-phenylvinyl]-8-quinolinol core structure that restored p53 levels *in vitro*, bound p53, induced p53 nuclear accumulation and target gene expression as well as caused non-genotoxic cell death. Furthermore, the compounds did not activate p53 by inhibiting the SCF-complex, by inhibiting proteasome degradation or by causing genotoxic stress. Finally, 2-[(E)-2-phenylvinyl]-8-quinolinol core structures bound to p53 with nanomolar affinities but required 10-fold higher concentrations to cause cytotoxicity. Higher concentrations necessary for cytotoxicity may also depend on the compound’s membrane permeability, stability and solubility in cell culture. Although loss of p53 partially reversed the cytotoxicity of core compounds, the higher compound concentrations necessary for cytotoxicity also suggests that these compounds may target both p53-dependent and p53-independent pathways. Thus, we have identified a new class of cytotoxic drugs that target p53 and possibly p53-independent pathways using a screening approach that can extend to identify novel inhibitors of other protein interactions.

To identify compounds that bound p53, we used a multiplexed reporter targeting p53 and Rb1 to SCF-mediated degradation. Although this assay may detect compounds that inhibit the SCF complex and/or downstream mediators of degradation, the multiplexed assay provided an internal control to select for compounds that restored p53 degradation and avoided non-specific proteolytic inhibition. Using this assay, we identified compounds that bound p53 and mediated cytotoxicity that was partially dependent on p53. Since loss of p53 only partially reversed the cytotoxicity of 2-[(E)-2-phenylvinyl]-8-quinolinol core compounds, this observation suggests that these compounds may also target additional p53-dependent and p53-independent pathways that has been shown with other molecules [21]. For example, these compounds may also target other p53-family members such a p63 or p73. Nevertheless, the extent to which core compounds target these additional p53-family members or other proteins is the focus of future studies.

Many technological and biological hurdles exist to identify novel small molecules that inhibit protein-protein interactions [22, 23]. While we used a cell-based screening assay, several groups have used *in vitro* and *in silico* screens to identify compounds that modulate protein-protein interactions [24–27]. Yet, *in vitro* assays such as ELISA based methods and AlphaScreen assays [24, 28] are prone to false positives due to “promiscuous hitters” that form aggregates and/or interfere with the protein chemistry resulting in a loss of signal. [27, 29, 30]. By contrast, we designed our assay to result in hits having a gain-of-signal in order to minimize false positive hits due to non-specific loss of signal. Furthermore, *in vitro* and *in silico* assays identified hits often required extensive chemical modifications to obtain cell permeable compounds. Our screen inherently selected for cell permeable compounds. In addition, cell-based protein-protein screens such as two-hybrid based assays [31, 32] are less frequently used likely due to false positive rates as high as 50% [33]. Again, these high false positive rates reflected assays designed for loss of signal to identify positive hits. To further optimize our assay, we engineered our multiplexed screen to provide both an internal specificity control as well as a second target for evaluation. Thus, with its increased screening efficiency, our unique high-throughput assay may better identify inhibitors of other protein-protein interactions.

Using this system, we have identified compounds containing a 2-[(E)-2-phenylvinyl]-8-quinolinol core structure that binds to and activates p53. Although only 24 of 160 structurally related compounds were active in our reporter assay, many of the inactive compounds had higher cLogP values suggesting that the lack of activity for some core compounds was related to their decreased solubility. Previously, work from Wang and others identified a 7-[anilino(phenyl)methyl]-2-methyl-8-quinolinol based compound that inhibited the MDM2-p53 interaction in pancreatic cancer cell lines [34, 35]. This 7-[anilino(phenyl)methyl]-2-methyl-8-quinolinol shares a similar 8-quinolinol moiety which further supports our conclusions that compounds containing a core 2-[(E)-2-Phenylvinyl]-8-quinolinol structure binds to and activates p53.

2-[(E)-2-Phenylvinyl]-8-quinolinol core structures are separate from previously identified p53 activators. Our data demonstrates that 2-[(E)-2-Phenylvinyl]-8-quinolinol structure binds to p53, induced p53 nuclear localization and transcription of p53 target genes indicating that compounds activated p53. Although another compound, RITA, has been shown to activate the p53 pathway, it remains unclear what extent RITA directly binds to p53 [18, 36]. In our degradation assay, RITA was not sufficient to stabilize the p53 reporter interaction suggesting our compounds likely stabilized p53 via a distinct mechanism. The majority of p53 stabilizing compounds, including 4,5-dihydroimidazoline (Nutlin) and spirooxindole-based molecules, bind to MDM2, E6 or E6AP in order to disrupt the aberrant degradation of p53 in cancers [37, 38]. By contrast, our compounds bound to p53 with nanomolar affinities while binding to the p53 binding partners E6 or E6AP with 10-fold less or no affinity. Other compounds, such as 2,2-bis(hydroxymethyl)-3-quinuclidinone (PRIMA-1), restored the function of mutant p53 likely by covalently binding to the p53 core domain [39, 40]. Similarly, our compound docked to the p53 core domain forming hydrogen bonds and bound to p53 with high affinities. Interestingly, 2-[(E)-2-Phenylvinyl]-8-quinolinol also induced p53 activation in C33a cells as detected by nuclear localization, promoter site binding, target gene transcription and cytotoxicity. Given that C33a cells possess a R273C mutation in p53, these results suggest that 2-[(E)-2-Phenylvinyl]-8-quinolinol core structures may also activate mutant p53, which is the subject of future work. Since molecular modeling suggests that these compounds bind to the DNA binding domain of p53, it is interesting to speculate that these compounds may alter the folding of the DNA binding domain, in part, to rescue the transcriptional activity of p53 mutants. Similarly, other compounds have been found to reactivate mutant p53 alleles by approximating a wild type p53 structure [41, 42]. Thus, we present a class of compounds that likely bind to p53, activate p53 pathways and induce cell death in part by a p53-dependent pathway.

For cancers, these core structures may represent a starting point to improve current therapies and minimize side effects by targeting oncogenic pathways present in malignant cells. Non-specific cytotoxic chemotherapies provide only marginal benefit in treating cancers, as cisplatin or other cytotoxic regimens in conjunction with radiation provided a 10 to 20% increase in progression free survival at the cost of toxicity rates that can be 5 to 10-fold higher than radiation alone [43–46]. In the metastatic setting, the best cytotoxic chemotherapies provided questionable benefit with response rates approximating 25%, median survivals of less than 12 months and no clear indication that chemotherapy prolongs life [47, 48]. By contrast, our compounds were as cytotoxic as current chemotherapies without the genotoxic stress associated with cisplatin. Consequently, future compounds may enable us to target cancer alone or concurrently with radiation and/or chemotherapy.

In conclusion, using a novel cell-based multiplexed protein targeted degradation assay, we have identified a core 2-[(E)-2-phenylvinyl]-8-quinolinol structure that inhibited degradation of p53. These compounds bind to p53 in order to restore p53 activity. Finally, these compounds induced cell death that was partially rescued by p53 suggesting that these compounds may also target additional pathways. Nevertheless, the activity of the 2-[(E)-2-Phenylvinyl]-8-quinolinol core structure may induce p53 pathways in general and, thereby, serve as molecular probes to further interrogate and potentially activate p53 function *in vitro* and *in vivo*. Furthermore, the novel screening system here may be readily applied to identify other inhibitors of protein-protein interactions including cell surface receptors, transcription factors and cell signaling pathways among others. Finally, the common structure presented here represents a potential starting point for optimizing new anti-neoplastic agents for cancers.

## Supporting Information

S1 Fig Compounds induced the p53 target gene expression. (A) Core compounds induced transcription of the p53 target genes PUMA, p21, BAX and FAS in HeLa cells. Cells were treated with the indicated compounds and, 8h later, the indicated gene expression was assessed by qRT-PCR using DDCT for GAPDH normalization. Results represent experiments performed in triplicate. Significance was assessed using the Student’s t-test. Error bars represent S.E.M. * indicates P < .05.
(PDF)

S2 Fig Loss of p53 partially rescued growth suppression by core compounds in HCT116 cells. wtHCT116 and mutHCT116 containing a null p53 allele were treated with Compound 4. Cell viability was assessed by Cell TiterGlo.
(PDF)

S3 Fig Compound 4 does not increase nuclear accumulation of p73. (A) wtHCT116 and mutHCT116 cells were treated with vehicle, cisplatin or compound 4 (cmpd 4) for 6h and cell lysates were subjected to nuclear fractionation. Lamin A/C marks the nuclear fraction.
(PDF)

S1 Table Primer Chart. (PDF)

S2 Table 2-[(E)-2-phenylvinyl]-8-quinolinol Core Structure restored p53. (PDF)

S3 Table p53 Modeling Results. (PDF)
