# Supplementary material for: A High-Throughput Cell-Based Screen Identified a 2-[(E)-2-Phenylvinyl]-8-Quinolinol Core Structure That Activates p53
Source: PLoS One. 2016 Apr 28;11(4):e0154125. doi: 10.1371/journal.pone.0154125 (PMC4849654; doi:10.1371/journal.pone.0154125)
Supplement: S1 Fig — (A) Core compounds induced transcription of the p53 target genes PUMA, p21, BAX and FAS in HeLa cells. Cells were treated with the indicated compounds and, 8h later, the indicated gene expression was assessed by qRT-PCR using DDCT for GAPDH normalization. Results represent experiments performed in triplicate. Significance was assessed using the Student’s t-test. Error bars represent S.E.M. * indicates P < .05. (PDF) [file pone.0154125.s001.pdf]

A

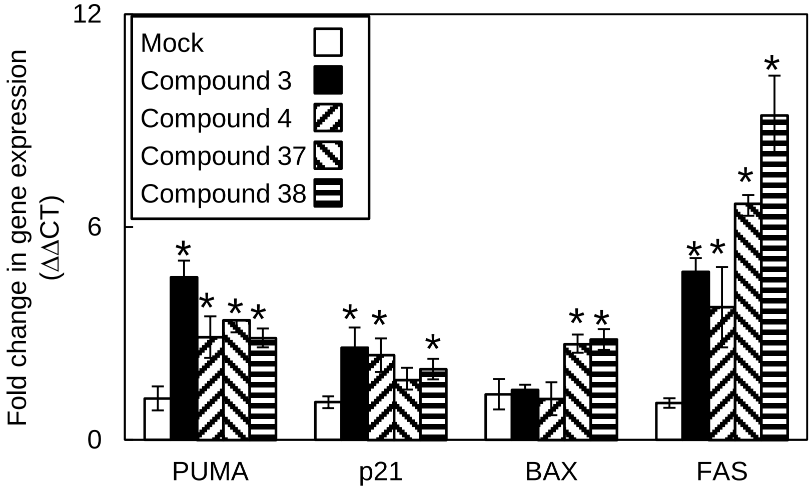

S1 Figure. Compounds induced the p53 target gene expression. (A) Core compounds induced transcription of the p53 target genes PUMA, p21, BAX and FAS in HeLa cells. Cells were treated with the indicated compounds and, 8h later, the indicated gene expression was assessed by qRT-PCR using ΔΔCT for GAPDH normalization. Results represent experiments performed in triplicate. Significance was assessed using the Student's t-test. Error bars represent S.E.M. * indicates P < .05.
