# Supplementary material for: A High-Throughput Cell-Based Screen Identified a 2-[(E)-2-Phenylvinyl]-8-Quinolinol Core Structure That Activates p53
Source: PLoS One. 2016 Apr 28;11(4):e0154125. doi: 10.1371/journal.pone.0154125 (PMC4849654; doi:10.1371/journal.pone.0154125)
Supplement: S2 Fig — wtHCT116 and mutHCT116 containing a null p53 allele were treated with Compound 4. Cell viability was assessed by Cell TiterGlo. (PDF) [file pone.0154125.s002.pdf]

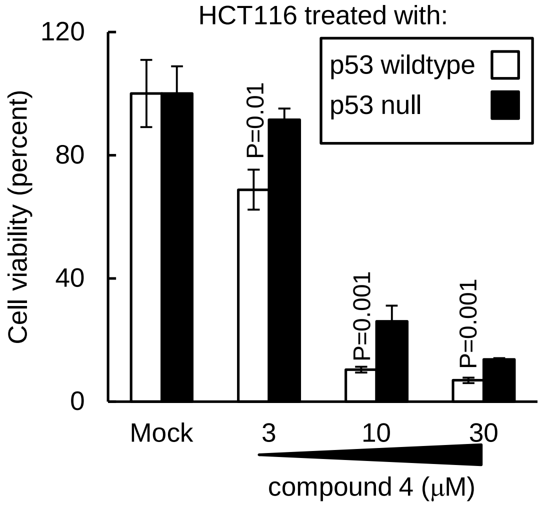

S2 Figure. Loss of p53 partially rescued growth suppression by core compounds in HCT116 cells. wtHCT116 and mutHCT116 containing a null p53 allele were treated with Compound 4. Cell viability was assessed by Cell TiterGlo.
