# Supplementary material for: A High-Throughput Cell-Based Screen Identified a 2-[(E)-2-Phenylvinyl]-8-Quinolinol Core Structure That Activates p53
Source: PLoS One. 2016 Apr 28;11(4):e0154125. doi: 10.1371/journal.pone.0154125 (PMC4849654; doi:10.1371/journal.pone.0154125)
Supplement: S3 Fig — (A) wtHCT116 and mutHCT116 cells were treated with vehicle, cisplatin or compound 4 (cmpd 4) for 6h and cell lysates were subjected to nuclear fractionation. Lamin A/C marks the nuclear fraction. (PDF) [file pone.0154125.s003.pdf]

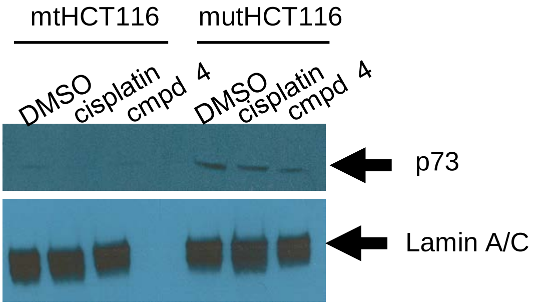

S3 Figure. Compound 4 does not increase nuclear accumulation of p73. (A) wtHCT116 and mutHCT116 cells were treated with vehicle, cisplatin or compound 4 (cmpd 4) for 6h and cell lysates were subjected to nuclear fractionation. Lamin A/C marks the nuclear fraction.
