# Supplementary material for: A High-Throughput Cell-Based Screen Identified a 2-[(E)-2-Phenylvinyl]-8-Quinolinol Core Structure That Activates p53
Source: PLoS One. 2016 Apr 28;11(4):e0154125. doi: 10.1371/journal.pone.0154125 (PMC4849654; doi:10.1371/journal.pone.0154125)
Supplement: S1 Table — (PDF) [file pone.0154125.s004.pdf]

S1 Table: Primer Chart

Primer name	Primer sequence	Description
p53 fwd primer	TTGAATTCGCCACCATGGAGGAGCCGCAGT	underline represents the EcoRI site, bold represents the KOZAK sequence
p53 rev primer	TTCTCGAGACCGGTGTCTGAGTCAGGCCCTTC	bold represents the AgeI site, underline represents the XhoI site
luc fwd primer	TTACCGGTGGCGGCGGCGGCGGCGGCGGCATGGAAGACGCCAAAAAC	bold represents the AgeI site, underline represents the poly-glycine linker
luc rev primer	TTCTCGAGTTACACGGCGATCTTTCC	underline represents the XhoI site
Rb fwd primer	TTGAATTCGCCACCATGAACACTATCCAACAAT	underline is the EcoRI site, bold is the KOZAK sequence
Rb rev primer	TTCTCGAGTTATTTCTCTTCCTTGTTTGAGGT	underlined represents the XhoI site
ren rev primer	TTACCGGTGGCGGCGGCGGCGGCGGCGGCATGACTTCGAAAGTTTATGATCC	
ren fwd primer	TTCTCGAGTTATTGTTCATTTTTGAGAACTCG	
TRCP fwd primer	TTCAATTGGCCACCATGGACTACAAGGACGACGATGACAAAATGGACCCGGCCGA	bold is MfeI site, underline is the FLAG site
TRCP rev primer	TTGGATCCACCGCCTCCAGAACCTCCTCCACCACTATGTCTTCCACATCTCCAATTAGATTC	bold is BamHI site, underline is the poly-glycine linker
E6 fwd primer	TTGGATCCGGTGGCGGAGGTAGCGGTGGCGGAGGTAGCTTTCAGGACCCACAGGAG	underline represents the poly-glycine linker, bold represents the BamHI sequence
E6 rev primer	TTCTCGAGTTACAGCTGGGTTTCTCTAC	underline represents the XhoI site
E7 fwd primer	TTGGATCCGGTGGCGGAGGTAGCGGTGGCGGAGGTAGCATGCATGGAGATACACCTACA	underline represents the poly-glycine linker, bold represents the BamHI sequence
E7 rev primer	TTCTCGAGTCAGGCTCTGTCCGGTTCTGCTTG	underline represents the XhoI sequence
GAPDH fwd primer	TGCACCACCAACTGCTTAGC	real-time primer
GAPDH rev primer	GGCATGGACTGTGGTCATGAG	real-time primer
PUMA fwd primer	CTCAACGCACAGTACGAG	real-time primer
PUMA rev primer	GTCCCATGATGAGATTGTACAG	real-time primer
