# Supplementary material for: A High-Throughput Cell-Based Screen Identified a 2-[(E)-2-Phenylvinyl]-8-Quinolinol Core Structure That Activates p53
Source: PLoS One. 2016 Apr 28;11(4):e0154125. doi: 10.1371/journal.pone.0154125 (PMC4849654; doi:10.1371/journal.pone.0154125)
Supplement: S2 Table — (PDF) [file pone.0154125.s005.pdf]

S2 Table: 2-[(E)-2-phenylvinyl]-8-quinolinol Core Structure restored p53

Compound	ID	Structure
**Hits from high-through-put screen**		
3	6634438	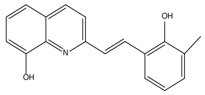
4	6636427	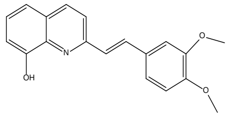
5	6637385	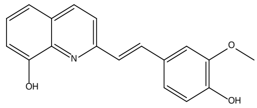
6	7007221	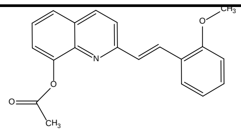
22	6635849	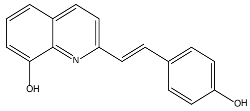
26	7007475	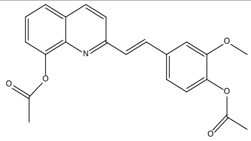
**Hits from SAR analysis**		
34	6634500	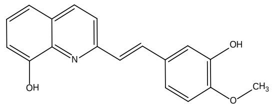
35	7021054	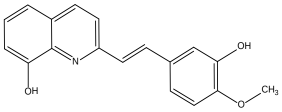
36	6373051	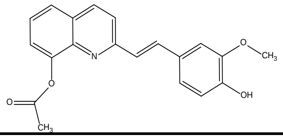
37	6637334	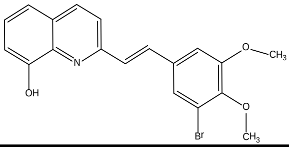

S2 Table: continued

Compound	ID	Structure
38	7350913	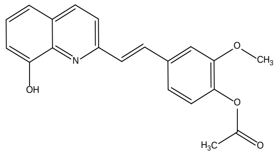
39	6376287	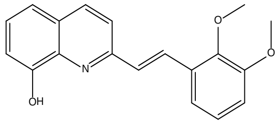
40	6634084	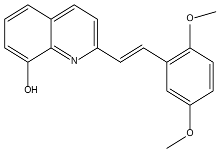
41	6634453	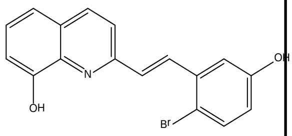
42	6635279	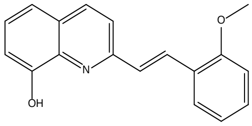
43	6636797	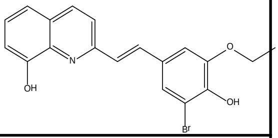
44	6636848	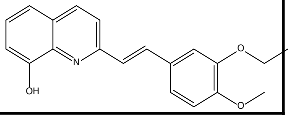
45	6636933	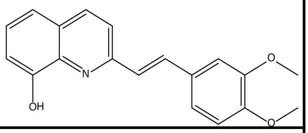
46	6637385	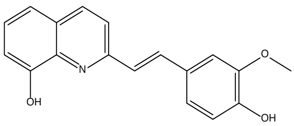
47	7002194	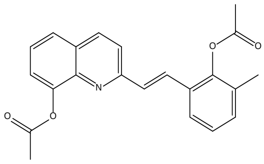

S2 Table: continued

Compound	ID	Structure
48	7007221	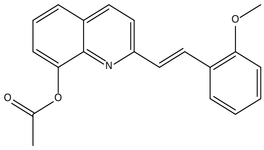
49	7018151	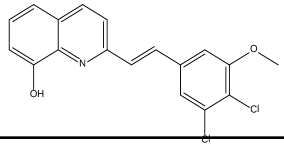
50	7018409	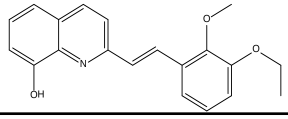
51	7319262	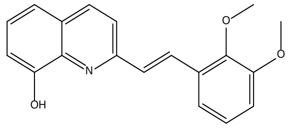
52	7326053	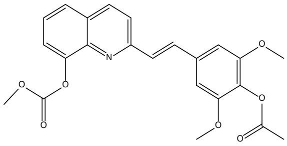
53	7326301	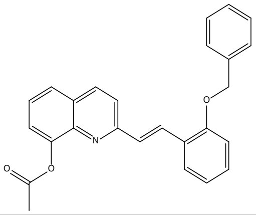
54	6372467	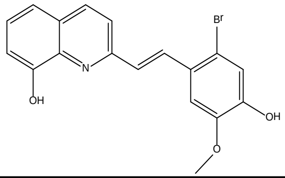
55	6376287	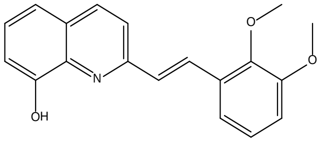
56	7319262	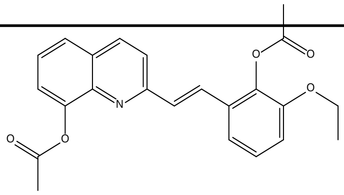
57	7326053	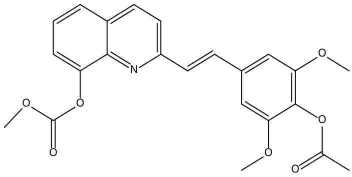
