# Supplementary material for: A High-Throughput Cell-Based Screen Identified a 2-[(E)-2-Phenylvinyl]-8-Quinolinol Core Structure That Activates p53
Source: PLoS One. 2016 Apr 28;11(4):e0154125. doi: 10.1371/journal.pone.0154125 (PMC4849654; doi:10.1371/journal.pone.0154125)
Supplement: S3 Table — (PDF) [file pone.0154125.s006.pdf]

S3 Table: p53 Modeling Results

Name	Compound ID	SAR score	Atoms	Weight	Flexible bonds	Score	Hydrogen bond score	Steric inter-action score	Ligand conformation penalty
Compound 11	ID 7002194	1.57	50	391.42	8	-44.36	-2.00	-50.26	7.90
Compound 36	ID 6373051	4.2	42	335.35	5	-44.22	-7.03	-46.80	9.60
Compound 31	ID 6636787	0.9	40	386.24	4	-43.57	-4.00	-41.91	2.34
Compound 10	ID 6637385	3.4	37	293.32	3	-42.72	-5.67	-38.28	1.24
Compound 34	ID 6634500	6.3	43	321.37	5	-42.28	-6.00	-39.95	3.67
Compound 4	ID 6636427	2.8	40	307.34	4	-42.21	-8.25	-37.73	3.77
Compound 35	ID 7021054	6.4	37	293.32	3	-42.01	-10.00	-38.57	6.56
Compound 38	ID 7350913	4	42	335.35	5	-41.52	-4.00	-42.37	4.85
Compound 6	ID 7007221	4.7	41	319.35	5	-41.33	-3.70	-43.30	5.67
Compound 37	ID 6637334	5	40	386.24	4	-40.85	-4.00	-41.41	4.56
Compound 3	ID 6634438	5.8	36	277.32	2	-40.08	-3.61	-42.13	5.66
Compound 30	ID 6636848	1.9	40	341.79	4	-39.55	-6.79	-36.95	4.18
